# Islets from human donors with higher but not lower hemoglobin A1c levels respond to gastrin treatment in vitro

**DOI:** 10.1371/journal.pone.0221456

**Published:** 2019-08-20

**Authors:** Ayelet Lenz, Gal Lenz, Hsun Teresa Ku, Kevin Ferreri, Fouad Kandeel

**Affiliations:** Department of Translational Research and Cellular Therapeutics, Diabetes and Metabolism Research Institute, Beckman Research Institute of City of Hope, Duarte, California, United States of America; University of Ulster, UNITED KINGDOM

## Abstract

Gastrin is a peptide hormone, which in combination with other factors such as TGFα, EGF or GLP-1, is capable of increasing beta cell mass and lowering blood glucose levels in adult diabetic mice. In humans, administration of a bolus of gastrin alone induces insulin secretion suggesting that gastrin may target islet cells. However, whether gastrin alone is sufficient to exert an effect on isolated human islets has been controversial and the mechanism remained poorly understood. Therefore, in this study we started to examine the effects of gastrin alone on cultured adult human islets. Treatment of isolated human islets with gastrin I for 48 h resulted in increased expression of insulin, glucagon and somatostatin transcripts. These increases were significantly correlated with the levels of donor hemoglobin A1_c_ (HbA_1c_) but not BMI or age. In addition, gastrin treatment resulted in increased expression of *PDX1*, *NKX6*.*1*, *NKX2*.*2*, *MNX1* and *HHEX* in islets from donors with HbA_1c_ greater than 42 mmol/mol. The addition of YM022, an antagonist of the gastrin receptor cholecystokinin B receptor (CCKBR), together with gastrin eliminated these effects, verifying that the effects of gastrin are mediated through CCKBR.CCKBR is expressed in somatostatin-expressing delta cells in islets from all donors. However, in the islets from donors with higher HbA_1c_ (greater than 42 mmol/mol [6.0%]), cells triple-positive for CCKBR, somatostatin and insulin were detected, suggesting a de-differentiation or trans-differentiation of endocrine cells. Our results demonstrate a direct effect of gastrin on human islets from prediabetic or diabetic individuals that is mediated through CCKBR^+^ cells. Further, our data imply that gastrin may be a potential treatment for diabetic patients.

## Introduction

After the first discovery that gastrin-expressing cells are found in the islets of Langerhans in rat embryos during the time of beta cell proliferation [1], many laboratories have explored the idea of employing gastrin to stimulate adult islets. It was shown that a bolus of gastrin administration enhances insulin secretion in humans [2, 3] and that human islets express the gastrin receptor, CCKBR [4]. Numerous publications showed a long-term gastrin effect on beta cell mass and glucose levels in diabetic murine models or models of pancreas injury. However, many of those papers indicated the need to combine gastrin treatment with additional factors such as TGFα, EGF or GLP-1 in order to affect beta cell mass and reduce glucose levels [5–10]. Inconsistent results have been observed when islets are treated with gastrin alone and the reason behind this inconsistency has not been well understood. In addition, the majority of the earlier studies were performed on rodent islets and very little is known about the effects of gastrin treatment on adult human islets. More recently, Dahan et al. described the expression of gastrin in a low percentage of delta and beta cells in islets of donors with type 2 diabetes but not in healthy adult islets [11], which were shown to express low levels of progastrin [12]. Yet, the function of gastrin in type 2 diabetic islets was not investigated by the authors.

An increasing body of evidence suggests that the loss of beta cell mass in type 2 diabetes is associated with de-differentiation of mature beta cells into a less-differentiated fetal state, or trans-differentiation of beta cells into other islet cell types [13]. In mouse models, deficient expression of essential beta cell transcription factors such as Pdx1, Nkx6.1, Nkx2.2, Mafa and Mnx1 results in diabetes, loss of beta cell identity and, in many cases, leads to the acquisition of phenotypes resembling either alpha or delta cells [14–18]. Loss of Hhex in islets also leads to delta cell de-differentiation and disrupted paracrine control on beta and alpha cells [19]. In human type 2 diabetic islets, the ratio of alpha to beta cell is increased compared to healthy individuals [20]. Whether other endocrine cells, such as delta cells, which express the CCKBR gastrin receptor [21], are also increased in islets of individuals with type 2 diabetes remains unknown. To address these questions, we investigated the effects of gastrin on adult human islets *in vitro*.

## Materials and methods

### Cell culture

Human islets were provided by the Southern California Islet Cell Resources Center (SC-ICRC) at City of Hope (Duarte, CA). The study was done on human islets approved for research use by the City of Hope Institutional Review Board and with the written informed consent from each organ donor family. Donor characteristics are listed in Table 1 Islet preparations were categorized by the donor’s HbA_1c_ level as either higher HbA_1c_ (>42 mmol/mol [6.0%]) or lower HbA_1c_ (<42 mmol/mol), out of the 13 donors with high HbA_1c_ levels, 6 were treated with oral medications for diabetes, for 2 donors it was unknown whether there was treatment and the rest were untreated. None of the donors were diagnosed with type 1 diabetes. Human Islets from 11 lower HbA_1c_ donors and 10 higher HbA_1c_ donors were received 2–4 days following isolation and incubated in 37 °C with 5% CO_2_ for 48 h in CMRL 1066 CIT Modifications medium (Corning, Corning NY, USA), containing 5.6 mmol/liter glucose, 2% human serum albumin (HSA) (Shire, Dublin, Ireland), 100 ng/ml, Insulin-like growth factor 1 (IGF-1) (Cell Sciences, Newburyport, MA, USA) and 10 nmol/l ol/l or 100 nmol/l [Leu15]-gastrin I (Sigma, St. Louis, MO, USA) for 48 h in order to avoid any loss of islet cells phenotype associated with long-term culturing [22–24]. YM022 (Sigma) a CCKBR antagonist, was added at a concentration of 100 nmol/l, in the absence or presence of 100 nmol/l gastrin to human islets isolated from 5 lower HbA_1c_ donors and 4 higher HbA_1c_ donors.

**Table 1:** List of islet donors.

Donor No.	Sex	Age	BMI	HbA1_C_ (mmol/mol)	HbA1_C_ (%)	T2D Oral Medication
1	F	63	27.5	27.0	4.6	No
2	M	48	25.7	29.0	4.8	No
3	F	50	21.5	30.0	4.9	No
5	F	35	28.7	30.0	4.9	No
6	F	40	36.0	31.0	5.0	No
7	M	52	21.0	31.0	5.0	No
8	M	15	24.0	32.0	5.1	No
9	F	55	34.8	33.0	5.2	No
10	M	37	30.2	34.0	5.3	No
12	F	57	26.0	36.0	5.4	No
13	M	59	30.8	36.0	5.4	No
14	F	48	31	37.0	5.5	No
15	M	46	33.2	37.0	5.5	No
16	F	65	35.1	38.0	5.6	No
17	M	57	23.0	39.0	5.7	No
18	M	60	31.18	39.0	5.7	No
19	M	63	25.6	41.0	5.9	No
20	M	62	27.5	42.0	6.0	No
21	M	49	34.1	43.0	6.1	No
22	M	57	30.6	44.0	6.2	No
23	F	49	29.2	45.0	6.3	No
24	M	45	36.4	49.0	6.6	Unknown
25	F	52	39.9	57.0	7.4	Yes-Metamorfin
26	M	62	35.9	57.0	7.4	Yes-Unknown
27	M	47	26.2	58.0	7.5	Yes-Metamorfin
28	M	62	30.1	62.0	7.8	Yes-Unknown
29	F	65	29.3	69.0	8.5	Yes-Unknown
30	M	44	42.3	75.0	9.0	Yes-Unknown
31	M	57	34.5	90.0	10.4	No
32	F	53	24.0	107.0	11.9	Unknown

### RNA isolation, cDNA synthesis and qPCR

Total RNA was extracted from purified isolated islets using the Direct-zol RNA MicroPrep Isolation Kit (Zymo, Irvine, CA, USA), and treated with RNase-free DNase1 (Thermo Fisher Scientific, Waltham, MA, USA). cDNA was produced using High-Capacity cDNA Reverse Transcription Kit (Applied Biosystems, Foster City, CA, USA). qPCR was carried out in a 7500 Real-time PCR system (Applied Biosystems) using the Assay-on-Demand kits (Applied Biosystems) listed in Table 2 All reactions were done in triplicate. Results were normalized to the transcripts of TATA-box-binding protein (TBP) and Ribosomal protein large P0 (RPLP0).

**Table 2:** Assay-on-Demand (Applied Biosystems) TaqMan fluorogenic probes used in the study.

Gene	Probe
*INS*	Hs02741908_m1
*GCG*	Hs01031536_m1
*SST*	Hs00356144_m1
*PDX1*	Hs00236830_m1
*MAFA*	Hs01651425_s1
*CCKBR*	Hs00176123_m1
*TBP*	Hs00427620_m1
*RPLP0*	Hs00420895_gH
*NKX6-1*	Hs00232355_m1
*NKX2-2*	Hs00159616_m1
*MNX1*	Hs00907365_m1
*HHEX*	Hs00242160_m1
*SOX5*	Hs00374709_m1
*BBC3*	Hs00248075_m1
*CASP2*	Hs00892481_m1
*CASP3*	Hs00234387_m1
*CASP8*	Hs01018151_m1
*CASP9*	Hs00962278_m1

### Immunofluorescence

Paraffin sections of pancreas from 7 lower HbA_1c_ donors and 8 higher HbA_1c_ donors were obtained from City of Hope islets isolation laboratory. Slides were de-paraffinized in xylene followed by re-hydration in ethanol. Antigen retrieval was done using Antigen Unmasking Solution (Vector laboratories, Burlingame, CA, USA). Samples were blocked for 20 min at room temperature in blocking buffer containing 1% BSA, 10% fetal Donkey serum, and 0.2% saponin (Sigma) and incubated overnight at 4°C with primary diluted in blocking buffer as follows: guinea pig anti-insulin (1:300, Dako, Santa Clara, CA, USA), rabbit anti-somatostatin (1:200, Dako), rat anti-somatostatin (1:50, R&D Systems, Minneapolis, MN, USA), mouse anti-glucagon (1:2000, Sigma), goat anti-CCKBR (1:200, Abcam, Cambridge, UK), and rabbit anti-CCKBR (1:100, Origene, Rockville, MD, USA). Slides were washed in PBS with 0.1% Tween (Sigma) 5 times and incubated with secondary antibodies conjugated to Alexa fluorophores (1:100, all from Jackson ImmunoResearch, West Grove, PA, USA,). DNA was stained with DAPI (Santa Cruz Biotechnology, Dallas, TX, USA). The slides were mounted with Fluorescent Mounting Medium (Dako). Images were visualized under a ZEISS inverted LSM 700 microscope using ZEN lite digital imaging software (Carl Zeiss, Oberkochen, Germany) for processing. To demonstrate specificity, a minus-primary antibody control was employed. The plugin JACoP of Image J was used to calculate the colocalization rate for the red and green signals as described [25]. Colocalization of signals from insulin (green) and somatostatin (red) was evaluated using Manders overlap coefficient and the Pearson’s correlation coefficient. The area on insulin and somatostatin were calculated using Image Pro.

### Statistical analysis

GraphPad Prism software was used to prepare data and analyze significance using 2-way ANOVA followed by a Tukey multiple comparison posttest to determine significance of differences between 3 groups or Sidak to determine significance of differences between 2 groups. For correlations between variables Pearson's correlation test was used. For colocalization analysis, the score for each channel was analyzed for individual islets and an unpaired t-Test was performed to compare the values obtained with islets from donors with HbA_1c_ > 42 mmol/mol to the values obtained with islets for donor with HbA_1c_ <42 mmol/mol for each channel separately. For analysis of insulin and somatostatin areas and the percentage of double positive cells an unpaired t-Test was performed to compare between islets from donors with HbA_1c_ >42 mmol/mol to islets from donor with HbA_1c_ <42 mmol/mol. Results were considered significant with p<0.05.

## Results

### Gastrin treatment changes gene expression in islets from donors with HbA_1c_ over 42 mmol/mol

**Fig 1:**
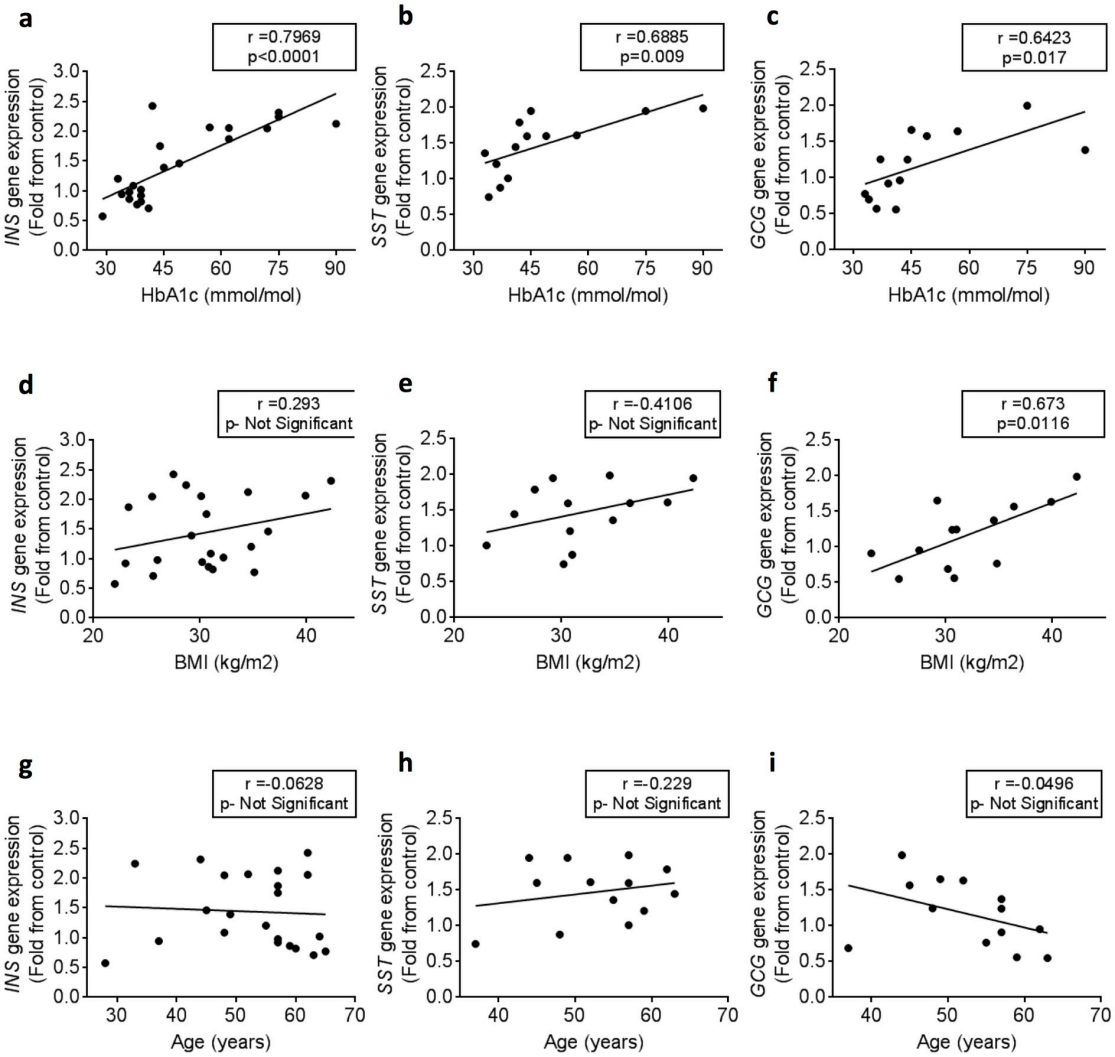
Correlation of donors HbA_1c_ levels with *INS*, *SST* and *GCG* genes fold change in response to gastrin treatment. Isolated human islets from a total of 13–22 independent donors were incubated with 0 nmol/l or 100 nmol/l gastrin for 48 h before qRT-PCR analysis a-c. Correlation of HbA_1c_ levels and *INS*, *SST* and *GCG* genes fold change in response to gastrin treatment. d-f. Correlation of donors BMI and *INS*, *SST* and *GCG* genes fold change in response to gastrin treatment. g-i. Correlation of donors age and *INS*, *SST* and *GCG* genes fold change in response to gastrin treatment. Pearson's correlation test was used to determine correlation and significance.

To investigate the effects of gastrin, islets from 22 donors were treated with 0 or 100 nmol/l gastrin for 48 h before qRT-PCR analysis. Pearson’s correlation analysis showed a significant correlation between the donors HbA_1c_ levels and fold change of *INS*, *SST* and *GCG* genes in response to 100nmol/l gastrin treatment (r = 0.79, p<0.0001; r = 0.69, p = 0.009; r = 0.64, p = 0.017 respectively) (Fig 1a to 1c). *GCG* fold change in response to gastrin treatment also significantly correlated with donors BMI (r = 0.673, p = 0.0116); however, *INS* and *SST* fold change did not (Fig 1d to 1f). None of fold change of these genes in response to gastrin treatment correlated with donors age (Fig 1g to 1i).

**Fig 2:**
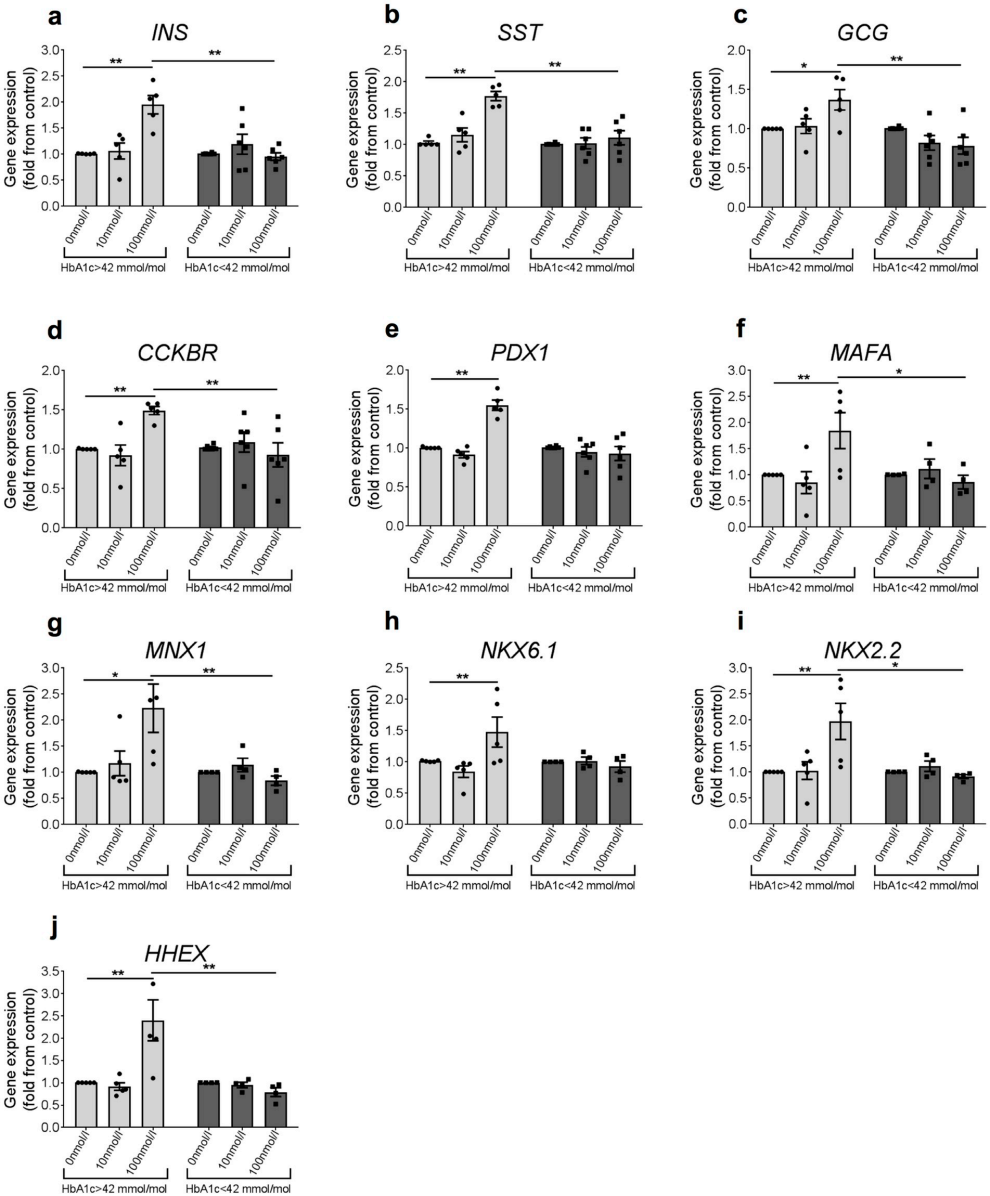
Gastrin enhances transcription of genes involved in islet cell function and identity in donors with higher HbA_1c_ levels. a-j. Isolated human islets were incubated with increasing doses (0, 10 and 100 nmol/l) of gastrin and cultured for 48 h before qRT-PCR analysis. Data represent mean ± SEM from a total of 5–6 independent donors of lower and higher HbA1c, respectively. A 2-way ANOVA followed by a Tukey multiple comparison posttest statistical analysis was performed to determine significance. * p<0.05, ** p<0.005.

To determine dose effect, islets from 11 donors, 5 with higher HbA_1c_ (HbA_1c_ >42 mmol/mol) and 6 with lower HbA_1c_ (HbA_1c_ <42 mmol/mol), were treated with 0, 10 or 100 nmol/l gastrin for 48 h before qRT-PCR analysis. In islets from donors with higher HbA_1c_, treatment with 100 nmol/l but not 10 nmol/l gastrin resulted in a 2-fold increase in *INS* expression, as well as increased expression of *SST* (1.8-fold), *GCG* (1.4-fold) and *CCKBR* (1.5-fold) (Fig 2a to 2j; left panels). Additionally, the expression of transcription factors essential for beta and delta cell development and function, such as *PDX1*, *MAFA*, *NKX6*.*1*, *NKX2*.*2*, *MNX1* and *HHEX*, were all significantly elevated in response to 100 nmol/l gastrin in islets from donors with higher HbA_1c_ levels. In contrast, in islets from donors with lower HbA_1c_, gastrin did not change the expression of these genes (Fig 2a to 2j; right panels). Islet cell survival was also examined following gastrin treatment; however, there were no significant changes in expression levels of genes related to apoptosis, such as *BBC3*, *CASP2*, *CASP3*, *CASP8* and *CASP9*, in islets from donors with either higher or lower HbA_1c_ levels and between islets treated with 100 nmol/l gastrin and control islets (S1a to S1e Fig).

### Gastrin confers its effect on transcription through CCKBR

**Fig 3:**
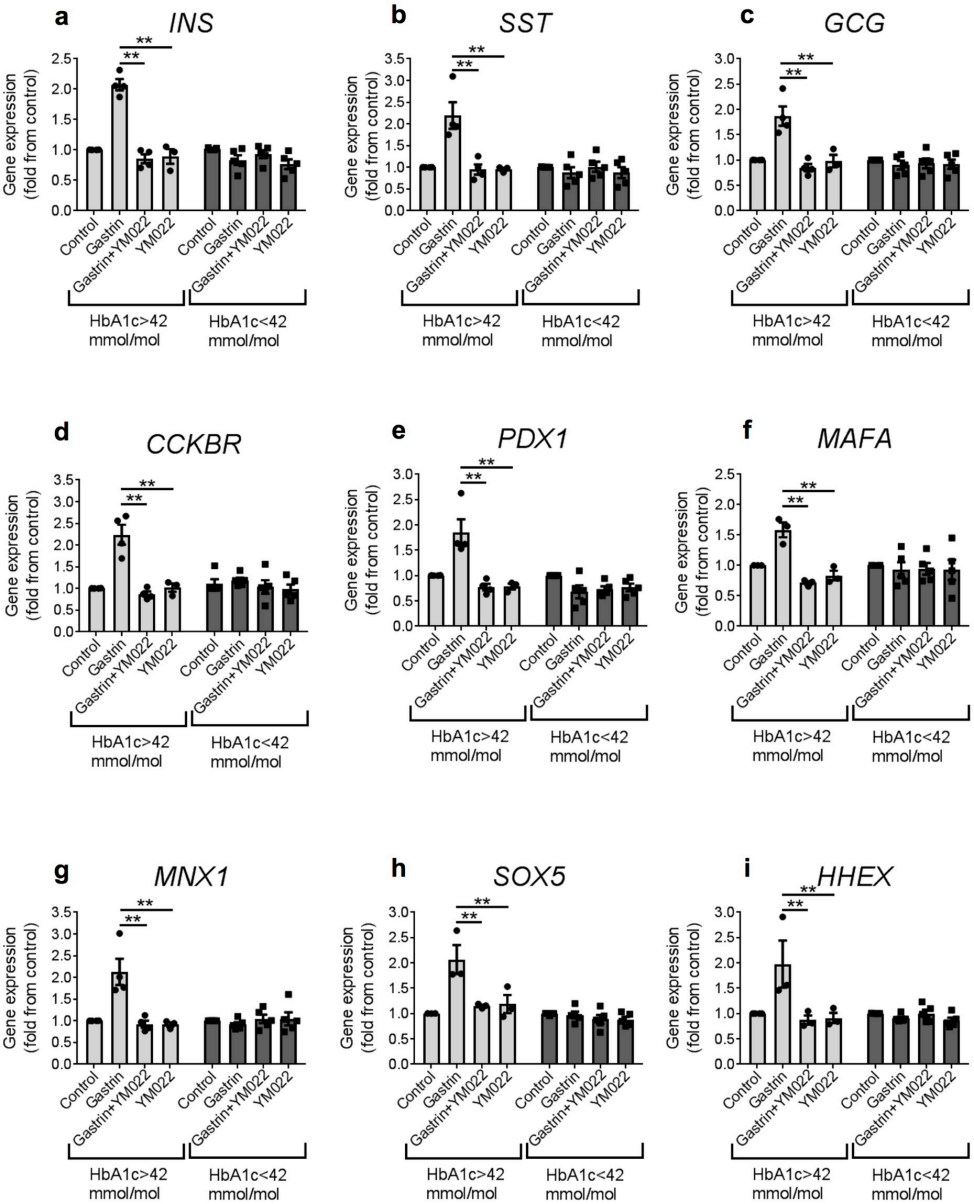
The effects of gastrin on human islets from donors with higher HbA_1c_ are mediated by the gastrin receptor CCKBR. a-i. Isolated human islets were incubated with 100 nmol/l gastrin and/or a CCKBR antagonist YM022, and cultured for 48 h before qRT-PCR analysis. Data are mean±SEM from a total of 5 and 3–4 independent donors of lower and higher HbA_1c_, respectively. A 2-way ANOVA followed by a Tukey multiple comparison posttest statistical analysis was performed to determine significance. * p<0.05, ** p<0.005.

To determine whether the effects of gastrin were mediated through CCKBR, islets were treated with YM022 (100 nmol/l), an extremely potent and highly selective antagonist to CCKBR [26, 27], in the presence of 100 nmol/l gastrin (n = 3–5). In this experiment, we also examined the expression of *SOX5* which plays a role in maintaining mature beta cells [28]. Consistent with previous results (Fig 2a to 2j), gastrin increased transcription of endocrine and developmental genes in islets from donor with higher HbA_1c_ (Fig 3a to 3i; left panels) but not lower HbA_1c_ (Fig 3a to 3i; right panels). Addition of YM022 in conjunction with gastrin prevented the increase in gene expression, suggesting that the effects of gastrin were mediated through CCKBR. Addition of YM022 alone to islets from all donors did not affect gene expression, suggesting that YM022 was not toxic to cells. Taken together, these data indicate that gastrin confers its effect through the gastrin receptor CCKBR.

### Protein expression of gastrin receptor CCKBR in human islets

**Fig 4:**
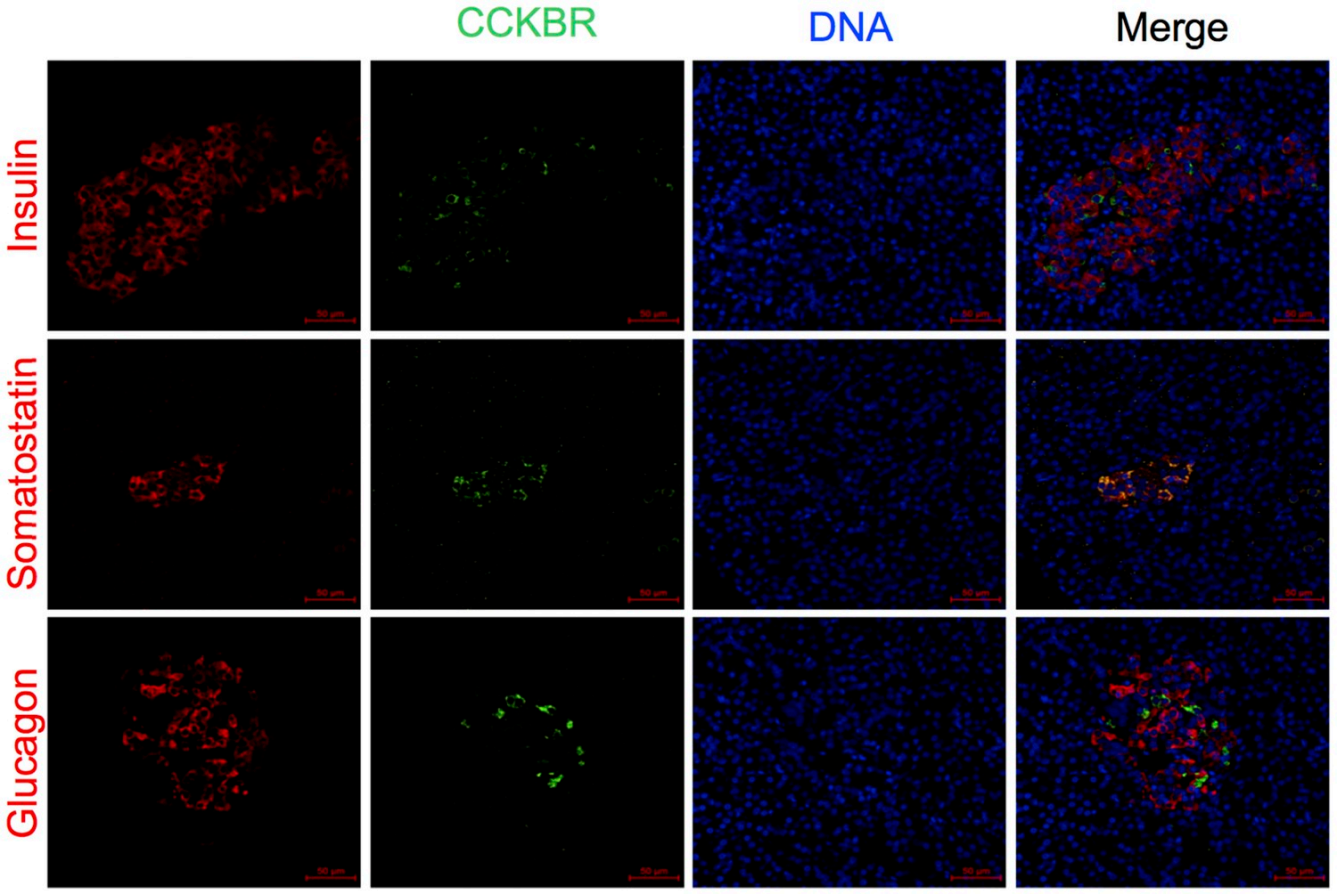
CCKBR is expressed in delta cells of islets from donors with HbA_1c_ < 42 mmol/mol. Formalin-fixed, paraffin-embedded tissue sections of adult human pancreases were examined by double immunofluorescence staining for gastrin receptor CCKBR (green) together with a major islet hormone; insulin, glucagon or somatostatin (red). DNA was stained blue with DAPI. Data from a total of 5–7 and 5–8 independent donors of lower and higher HbA_1c_, respectively.

**Fig 5:**
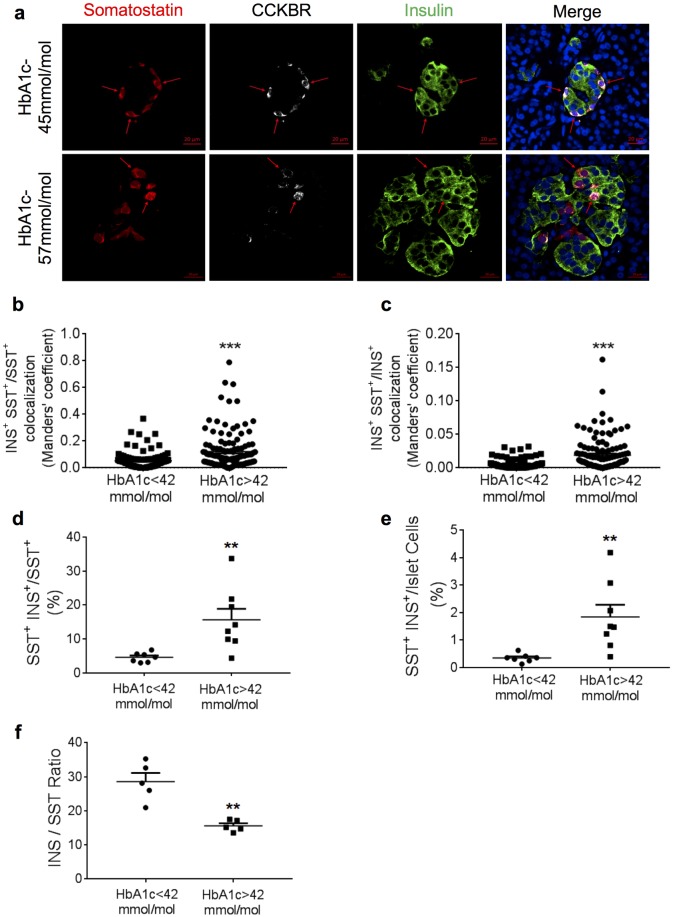
Cells simultaneously expressing insulin, somatostatin and CCKBR are more frequenly found in islets of donors with higher (>42 mmol/mol) HbA_1c_. a. Formalin-fixed, paraffin-embedded tissue sections of adult human pancreases were examined by triple immunofluorescence staining. Shown are examples of islet cells from donors with higher HbA_1c_ levels co-expressing insulin (green), somatostatin (red) and CCKBR (white). DNA was stained blue with DAPI. b. ratio of double-positive pixels for insulin and somatostatin over the sum of insulin-positive pixels. c. ratio of double positive pixels over the sum of somatostatin positive pixels. d. Percentage of insulin -positive cells among somatostatin -expressing cells is increased in islets of donors with higher HbA_1c_. Based on counting 102–400 delta-cells per donor. e. Percentage of cells expressing both insulin and somatostatin among the total number of islet cells is increased in islets of donors with higher HbA_1c_. Based on counting 1016–4022 cells per donor. f. Ratio between insulin positive area and somatostatin positive area was decreased in islets of donors with higher HbA_1c_. Data represent mean±SEM from a total of 5–7 and 5–8 independent donors of lower and higher HbA_1c_, respectively. Based on analyzing 18–30 islets per donor. An unpaired t-Test statistical analysis was performed to determine significance. ** p<0.005, *** p<0.0005.

To determine which cell type in the human islet responds to gastrin treatment, protein expression patterns of the gastrin receptor CCKBR was determined using immunofluorescence staining of human pancreas sections. In islets of donors with lower HbA_1c_, CCKBR was present in somatostatin expressing cells and absent in cells expressing insulin or glucagon (Fig 4), indicating that in healthy human islets CCKBR is located in delta cells. In contrast, in islets from donors with higher HbA_1c_ levels, cells triple positive for somatostatin, CCKBR and insulin were detected (Fig 5a). Further analysis of the red (somatostatin) and green (insulin) pixels in images showed an increase in the overlap and correlation between insulin and somatostatin signals in islets from donors with higher HbA_1c_ compared to islets from lower HbA_1c_ donors (Fig 5b and 5c). These results indicate an increase in the appearance of triple positive cells in donors with higher HbA_1c_. To verify, we counted insulin-expressing cells among total somatostatin positive cells, and among the total number of islet cells, and observed a significant increase in the percentage of double hormonal cells in donors with higher HbA_1c_ (Fig 5d and 5e). Additionally, the ratio between insulin area and somatostatin area was significantly decreased in islets from donors with higher HbA_1c_ due to an increased somatostatin positive area and not a decrease in insulin positive area (Fig 5f and S2a and S2b Fig).

## Discussion

In this study, we investigated the effects of exogenous gastrin on adult human islets in vitro. We discovered that in islets isolated from donors with HbA1_c_ higher than 42 mmol/mol, gastrin enhanced the expression of genes crucial for function and identity of islet cells, such as the islet hormones insulin, somatostatin, and glucagon, as well as important islet transcription factors. The cutoff of 42 mmol/mol was based on the Pearson’s correlation analysis (Fig 1a–1c). Additionally, we showed that the effects of gastrin on human islets were specifically mediated through the gastrin receptor, CCKBR, suggesting a direct action of gastrin on human islets.

To the best of our knowledge, we believe that the current study is the first to report the effects of gastrin treatment alone on isolated adult human islets based on the HbA1_c_ level of donors. Prior publications did not mention the HbA1_c_ levels of islet donors and it was assumed that those studies were done on islets isolated from healthy donors. For example, Suarez-Pinzon et al. treated adult human islets in vitro with gastrin and EGF and found an increase in beta cell mass [6]. Notably, this study and their follow-up study [7] were performed on dissociated human islet cells, a manipulation that is known to induce islet cell de-differentiation [29]. Thus, our results point to a need to stratify human islets using HbA1_c_ levels in future studies on gastrin.

It is also important to note that cholecystokinin-8 (CCK-8) binds to CCKBR with a similar affinity to that of gastrin [30]. Interestingly, previous studies demonstrated that during stress states such as obesity, islets produce and secret cholecystokinin (CCK) [31, 32], this was shown to have a positive effect on beta cell mass [29, 30]. Further investigation is needed in order to determine if the HbA1_c_ correlated responsiveness to exogenous gastrin is linked to this increase in CCK production within the islets during obesity which is known to correlate with increased HbA1_c_1 [33].

A new finding from the current study is the existence of triple-positive cells that simultaneously express insulin, somatostatin, and CCKBR in human islets from donors with higher HbA_1c_ levels. As mentioned, the presence of polyhormonal cells such as insulin and glucagon double-positive cells in islets of type 2 diabetic human donors [34, 35] has been associated with de-differentiation or trans-differentiation of beta cells [13]. Cells co-expressing insulin and somatostatin were shown in mouse models in which the expression of important beta-cell transcription factors such as *Nkx2*.*2*, *Nkx6*.*1* and *Mnx1* were deleted [15, 17, 18]. Decreased expression of these transcription factors and other important islet cell transcription factors was associated with human islet dysfunction and type 2 diabetes [34, 36]. Using single-cell RNA sequencing followed by immunofluorescence analysis, Teo et al. [37] recently reported the existence of cells co-expressing insulin and somatostatin in adult human islets and these cells were more abundant than insulin and glucagon co-expressing cells. This might be explained by the closeness of beta cells and delta cells in their lineage since both cell types are known to originate from the same PAX4^+^/ARX^-^ progenitor cell while alpha cells originate from PAX4^-^/ARX^+^ cells [38, 39]. However, nearly all prior studies on single cells from dissociated human islets disregard data containing double-hormones, assuming that they were the result of cell doublets [40–42].

Our results are consistent with the aforementioned literature and suggest that the triple-positive cells observed in our study may be a result of de-differentiation or trans-differentiation of beta-cells due to the natural progression of diabetes in donors with higher HbA_1c_ levels. Because gastrin enhances the expression of transcription factors involved in maintaining beta and delta cell identities (Figs 1 and 2), we also speculate that gastrin may stimulate the triple-positive cells to reverse back into somatostatin^-^insulin^+^ beta cells or force those triple-positive cells to resolve into a somatostatin^+^/insulin^-^ delta-cell state. The triple-positive cells are not absent from donors with lower HbA_1c_ levels (Fig 5). We therefore cannot rule out the possibility that gastrin treatment may also have an effect on islets from donors with lower HbA_1c_ levels, however, the effect on mRNA levels might be too small to detect when performing qPCR on the entire islet cell population. The reversibility of de-differentiated or trans-differentiated beta cells from type 2 diabetic islets into a normal state has been demonstrated in several murine models [43, 44]. However, further studies are required to demonstrate the reversibility and de-differentiation/trans-differentiation in adult type 2 diabetic human islets by studying the responses to gastrin from purified endocrine cell types or studies on the single-cell level, instead of the whole islets used in the current study.

It is also important to mention that CCKBR is thought to be found only in delta cells of human islets [21]; however, in the past CCKBR expression was reported in alpha cells as well [45]. Considering the impact of gastrin on human islets and the low percentage of delta cells within the islets, even in islets from donors with high HbA1_c_ levels, we cannot rule out the possibility that alpha cells might express a different isoform of CCKBR not detected by the antibodies commonly used today. It is of interest to observe that BMI of pancreas donors positively correlated with fold increase in gene expression of glucagon but not insulin or somatostatin under gastrin treatment (Fig 1f). The significance of such observation is not apparent from the existing literature but is currently under active investigation in our laboratory.

One potential confounding factor on the high HbA_1c_ donor islets was the fact that some of those donors had received medications, which may enhance responsiveness to gastrin. Additionally, it was shown that some anti-hyperglycemia drugs might improve beta-cell function and mass [46–48] and thus might affect gene expression in islet cells. However, an effect of medications is unlikely because we did not find differences in gene expression in response to gastrin treatment between islets isolated from donors treated with and without medications (not shown), in addition to the significant increase in important beta-cell gene expression in gastrin treated islets isolated from higher HbA1c donors compared to untreated islets. However, the possibility of anti-hyperglycemia drugs influencing the response to gastrin treatment should be further investigated. Another potential confounding factor was that *ex vivo*-cultured, rather than endogenous islets were studied. These cultured islets may have higher propensity to de-differentiate [37]. However, such a scenario should affect the baseline levels of de-differentiation and does not explain the differential effects of gastrin on high versus low HbA_1c_ islets. Future prospective studies in type 2 diabetic subjects are needed to directly assess long-term effects of exogenous gastrin on glycemic control and beta cell mass.

On this note, a suggested effect of gastrin has been implicated in patients with upper gastrointestinal disorders who were treated with proton pump inhibitors (PPIs) inducing endogenous gastrin secretion [49–53]. In type 2 diabetic patients, most retrospective and clinical studies showed an improved glycemic control and a decrease in HbA1_c_ levels by PPIs [54–56], although some showed limited success [57, 58]. Additionally, in murine models of type 2 diabetes, PPIs were shown to improve glycemic control [59].

In summary, we have shown a positive effect of exogenous gastrin treatment on the expression of genes important for islet cell function and identity in adult human islets isolated from donors with higher HbA_1c_ levels. This effect of gastrin is mediated through the gastrin receptor CCKBR. Taken together, our results support the notion of employing gastrin as a possible treatment for patients with type 2 diabetes.

## Supporting information

S1 Fig Gastrin does not induce cells death in treated islets. a-e. Isolated human islets were incubated with 0 nmol/l or 100 nmol/l gastrin and cultured for 48 h before qRT-PCR analysis. Data represent mean ± SEM from a total of 5 independent donors of lower HbA_1c_ and 5 independent donors of higher HbA_1c_. A 2-way ANOVA followed by a Sidak multiple comparison posttest statistical analysis was performed to determine significance.
(TIF)

S2 Fig Percentage of somatostatin positive area in islets of donors with higher (>42 mmol/mol) HbA_1c_ is increased while insulin positive area remains unchanged. Formalin-fixed, paraffin-embedded tissue sections of adult human pancreases were examined by immunofluorescence staining. a. Somatostatin positive area (%) is increased in islets from higher HbA_1c_ donors. b. Insulin positive area (%) is similar between higher and lower HbA_1c_ islet donors. Data represent mean±SEM from a total of 5 and 5 independent donors of lower and higher HbA_1c_, respectively. Based on analyzing 18–30 islets per donor. An unpaired t-Test statistical analysis was performed to determine significance. ** p<0.005.
(TIF)
