# Supplementary figures and images for: Islets from human donors with higher but not lower hemoglobin A1c levels respond to gastrin treatment in vitro
Source: PLoS One. 2019 Aug 20;14(8):e0221456. doi: 10.1371/journal.pone.0221456 (PMC6701795; doi:10.1371/journal.pone.0221456)

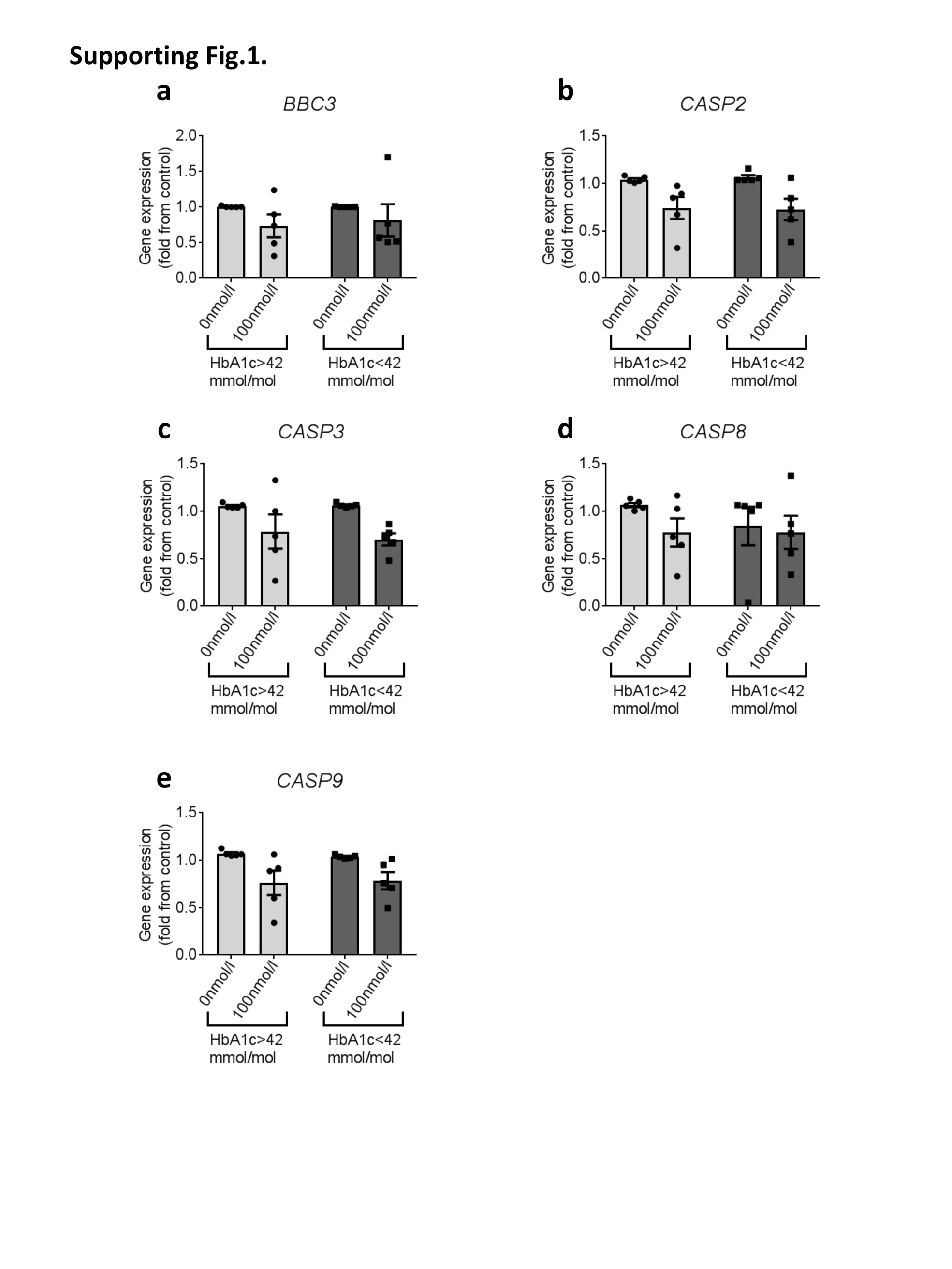

Supplement: S1 Fig — a-e. Isolated human islets were incubated with 0 nmol/l or 100 nmol/l gastrin and cultured for 48 h before qRT-PCR analysis. Data represent mean ± SEM from a total of 5 independent donors of lower HbA1c and 5 independent donors of higher HbA1c. A 2-way ANOVA followed by a Sidak multiple comparison posttest statistical analysis was performed to determine significance. (TIF) [file pone.0221456.s001.tif]

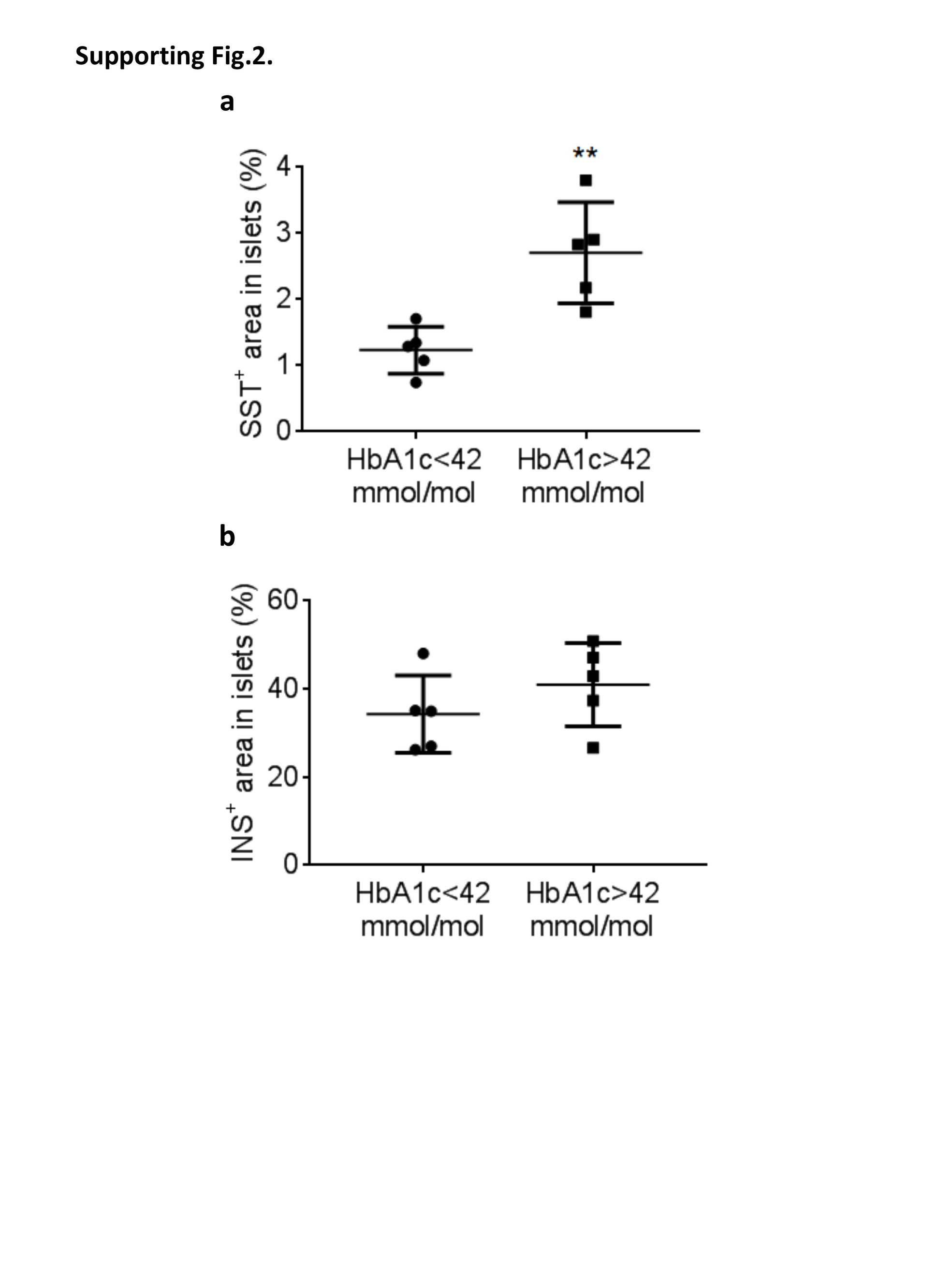

Supplement: S2 Fig — Formalin-fixed, paraffin-embedded tissue sections of adult human pancreases were examined by immunofluorescence staining. a. Somatostatin positive area (%) is increased in islets from higher HbA1c donors. b. Insulin positive area (%) is similar between higher and lower HbA1c islet donors. Data represent mean±SEM from a total of 5 and 5 independent donors of lower and higher HbA1c, respectively. Based on analyzing 18–30 islets per donor. An unpaired t-Test statistical analysis was performed to determine significance. ** p<0.005. (TIF) [file pone.0221456.s002.tif]
